# Novel compound heterozygous *PKHD1* mutations in a Chinese ARPKD pedigree and analysis of genotype-phenotype correlations

**DOI:** 10.3389/fmed.2026.1741795

**Published:** 2026-02-06

**Authors:** Jie Zhao, Kang Yu, Yujuan Huang

**Affiliations:** 1 Department of Emergency, Shanghai Children’s Hospital, School of Medicine, Shanghai Jiao Tong University, Shanghai, China; 2 Department of Second Dental Center, Ninth People’s Hospital Affiliated with Shanghai Jiao Tong University, School of Medicine, Shanghai Key Laboratory of Stomatology, National Clinical Research Center of Stomatology, Shanghai, China

**Keywords:** autosomal recessive polycystic kidney disease, genotype-phenotype, novel mutation, pathogenic mechanism, PKHD1

## Abstract

**Background:**

Autosomal recessive polycystic kidney disease (ARPKD) is an inherited renal disorder characterized by multiple renal cysts. This study aimed to investigate the pathogenesis of *PKHD1* gene variants in a Chinese ARPKD pedigree and elucidate the mechanisms underlying the phenotypic heterogeneity in patients with *PKHD1* mutations.

**Methods:**

Clinical data and blood samples were collected from the proband and family members. Whole-exome sequencing (WES) and Sanger sequencing were performed. Conservative analysis and local secondary structure prediction of the mutation site were performed to evaluate the pathogenicity. The genotype-phenotype correlation of PKHD1 mutations was analyzed in combination with this pedigree.

**Results:**

A pediatric patient with ARPKD was identified. Ultrasonography revealed bilateral renal enlargement with multiple cysts, accompanied by hepatic fibrosis. WES identified a novel compound heterozygous variant in the *PKHD1* gene (c.5850_5851insTTCAT; p.Gly1951Phefs*25 and c.8710G > A; p.Glu2904Lys). Conservation analysis confirmed that both mutations occurred in conserved regions, indicating potential pathogenicity. Secondary structure prediction revealed that the p.Gly1951Phefs*25 frameshift mutation resulted in protein truncation and conformational changes, whereas the p.Glu2904Lys missense mutation caused drastic changes in amino acid polarity, impairing protein stability and function. Genotype-phenotype analysis of 605 *PKHD1* mutations revealed a trend suggesting that hepatobiliary manifestations might present with less severe mutational burden compared to renal phenotypes, and mild homozygous missense mutations or heterozygous states may attenuate or eliminate renal phenotypes.

**Conclusion:**

This study uncovered novel PKHD1 mutations in an ARPKD patient, expanding the pathogenic gene spectrum of ARPKD and providing insights for genetic counseling and prenatal diagnosis. Our findings also contribute to the understanding of genotype-phenotype correlations in PKHD1 mutation carriers and generate hypotheses regarding potential organ-specific thresholds for disease manifestation.

## 1 Introduction

Polycystic kidney disease (PKD) is an inherited disorder primarily affecting the kidneys, characterized by the presence of multiple cysts in the renal cortex and medulla, often accompanied by polycystic lesions in the hepatobiliary system (1). Based on the inheritance patterns, PKD can be classified into autosomal recessive polycystic kidney disease (ARPKD, OMIM#263200) and autosomal dominant polycystic kidney disease (ADPKD, OMIM#601313) (2). ARPKD is a rare autosomal recessive monogenic cystic disorder with a global incidence of approximately 1/20,000. Clinically, it manifests as bilateral cystic kidneys and hepatic fibrosis, representing one of the most severe diseases leading to end-stage renal disease in children.

The primary pathogenic genes of ARPKD are polycystic kidney and hepatic disease 1 (PKHD1) and DAZ-interacting protein 1-like (DZIP1L). These genes encode the fibrocystin/polyductin (FPC) and DZIP1L proteins, both of which localize to cilia and are associated with ciliary defects; thus, ARPKD is classified as a ciliopathy (2–4). ARPKD is predominantly caused by PKHD1 mutations, whereas DZIP1L mutations are rare. The PKHD1 gene, located on chromosome 6p12, comprises 86 exons and encodes multiple protein isoforms, with FPC being the longest (5). FPC consists of 4074 amino acids and contains two core components: an N-terminal extracellular domain harboring IPT, PA14, G8, and PBH1 domains, and a C-terminal intracellular domain with a ciliary targeting sequence. This sequence may facilitate interactions between FPC and intracellular proteins such as polycystic protein-2 and can be released via Notch-like proteolytic cleavage to execute specific functions (5–7). FPC expression exhibits both tissue specificity and cell type specificity, and is found predominantly in ductal cells and their precursors in the developing kidney, liver, and pancreas (8, 9). Currently, the precise function of FPC remains incompletely understood, and it is hypothesized that FPC acts mainly as a receptor protein, potentially regulating ductal cell formation, proliferation, apoptosis, adhesion, and signal transduction (8).

The typical clinical manifestations of ARPKD in fetuses/neonates include increased bilateral renal volume in late pregnancy or the neonatal period, poor differentiation of the renal cortex and medulla, oligohydramnios, and pulmonary hypoplasia (10). Over 50% of ARPKD neonates die from respiratory insufficiency due to pulmonary hypoplasia. However, survival rates improve significantly beyond the perinatal period, with 1-year and 10-years survival rates reaching 85% and 82%, respectively (2). Owing to cyst formation and interstitial fibrosis in the renal distal tubules and collecting ducts, renal function progressively deteriorates, with approximately 50% of patients developing chronic renal failure in adulthood (5). The liver manifestations primarily include congenital hepatic fibrosis and portal hypertension (11). ARPKD can traditionally be clinically diagnosed in prenatal, neonatal, or end-stage patients exhibiting polycystic kidney features on ultrasound. However, these findings are non-specific and may overlap with those of other disorders, necessitating genetic confirmation (12, 13). Next-generation sequencing, particularly whole-exome sequencing (WES), has been widely applied in the diagnosis of monogenic disorders because of its broad sequencing coverage and relatively low cost, providing a basis for clinical genetic counseling (14, 15).

In this study, WES was employed to investigate the pathogenic mechanism of an ARPKD pedigree complicated with hepatic dysfunction, followed by a systematic genotype-phenotype analysis of PKHD1 gene mutations. These findings not only expand the mutation spectrum of the PKHD1 gene but also elucidate the genotype-phenotype correlation in PKHD1 mutation carriers, laying a foundation for more precise individualized diagnosis and treatment strategies.

## 2 Materials and methods

### 2.1 Subject

This study included one patient with ARPKD at Shanghai Children’s Hospital. The patient underwent clinical and imaging examinations conducted by specialists, including inquiries about family history, systemic disease history, examination of kidney and liver conditions, and recording of findings. Both the patient (or the guardian if the patient was under 18 years old) and their family members were informed of the content. The study was conducted in accordance with the principles of the Declaration of Helsinki. This study was approved by the Ethics Committee of Shanghai Children’s Hospital.

### 2.2 DNA sample collection

Peripheral venous blood (3–5 ml) was collected from the proband and family members, anticoagulated with EDTA, transferred into a labeled freezing tube, and stored at −80 °C.

### 2.3 Whole-exome sequencing

Blood genomic DNA was extracted from the proband and their immediate family members via a blood genomic extraction kit (Qiagen) according to the kit’s instructions. After DNA quality testing, exome capture, and library construction, quality control was performed on the library. Once the library passed quality inspection, whole-exome sequencing was conducted on the samples via the Illumina HiSeq PE150 sequencing platform. Bioinformatics analysis was performed on the raw sequencing data, including sequence alignment and variant detection; variant annotation based on variant information, disease information, population frequency, software prediction results, and pathogenicity reports; and variant filtering based on biological harmfulness, genetic coincidence (consistent with familial cosegregation, i.e., different phenotypes and genotypes between patients and healthy family members), and clinical feature coincidence. Mutations with a frequency greater than 0.01 in the Exome Aggregation Consortium (ExAC) and 1000 Genome Browsers (1000G) databases were removed, as well as mutations in intron regions, retaining only exon region mutations. Silent mutations that did not alter the amino acid sequence were also excluded. According to the guidelines of the American College of Medical Genetics and Genomics (ACMG),^1^ variants were classified as pathogenic, likely pathogenic, uncertain significance, likely benign, or benign.

### 2.4 Sanger sequencing and segregation analysis

Sanger sequencing was performed to verify the *PKHD1* (RefSeq: NM_138694.4) mutations we found. All sequence information was extracted from the NCBI database. The primers were designed using Primer 3^2^. SnapGene software was used to analyze the sequencing data. Familial cosegregation analysis was conducted on the probands and their family members, and variants that conform to phenotypic cosegregation were annotated as PP1 in the ACMG classification.

### 2.5 Conservation and structural modeling of the *PKHD1* variants

For conservation analysis, the amino acid sequence encoded by the human *PKHD1* gene was obtained from the UniProtKB database^3^. Strap^4^ was used to conduct the multiple sequence alignment. For tertiary structural analysis, the protein structure encoded by the *PKHD1* gene was predicted using AlphaFold3^5^ to analyze the impact of mutations on protein structure. All the crystal structure figures were generated using the PyMOL Molecular Graphics System^6^.

### 2.6 Genotype-phenotype analysis

Literature search was conducted in PubMed for articles reporting *PKHD1* variants and associated phenotypes published until April 31, 2023. Variants were included if they were explicitly linked to a clinical diagnosis in the report. Phenotypes were categorized based on the authors’ original descriptions as follows: ARPKD (bilateral renal cysts with or without hepatobiliary involvement); Caroli Disease (CD: isolated segmental intrahepatic bile duct dilatation) (16); Polycystic Liver Disease (PCLD: isolated multiple hepatic cysts) (17); Von Meyenburg Complexes (VMC) (18). For cases with longitudinal data, the most severe or latest reported phenotype was used. Cases with insufficient clinical detail or ambiguous diagnoses were excluded. This curation aims to summarize reported associations rather than calculate epidemiological risk.

## 3 Results

### 3.1 Clinical phenotype of the patient

**FIGURE 1:**
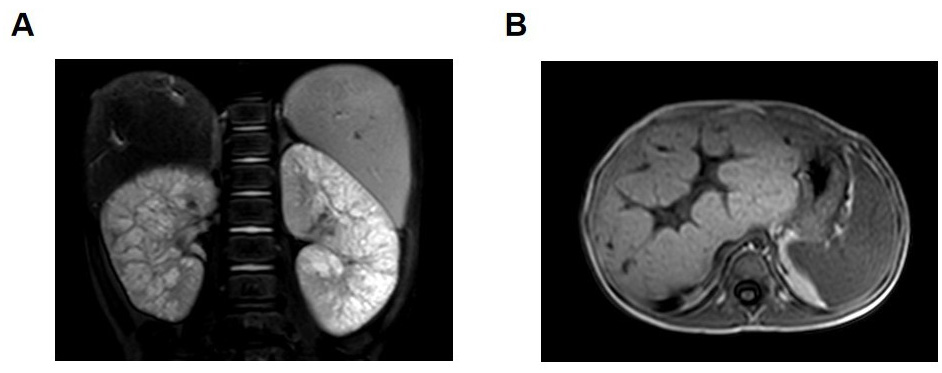
Magnetic resonance imaging (MRI) manifestations of the kidney and liver in a child with polycystic kidney disease. **(A)** Enlarged bilateral kidneys with multiple cysts. **(B)** Enlarged liver, disproportionate liver lobe ratio, and widened portal vein. MRI, magnetic resonance imaging.

A 5-years-old male patient was admitted for further evaluation following a 5-days history of bilateral lower extremity rash upon referral. The patient had a medical history of premature birth requiring neonatal hospitalization. Abdominal ultrasonography at 2 years of age revealed bilateral nephromegaly. Physical examination upon admission demonstrated hepatomegaly with a firm liver palpable 4 cm below the right costal margin and 6 cm below the xiphoid process. Splenomegaly was also noted, with the spleen palpable 3 cm below the left costal margin and exhibiting firm consistency. No lower extremity edema was observed. Relevant laboratory tests showed significant hepatic dysfunction, manifesting as jaundice, and the urine test results were not significant. Abdominal magnetic resonance imaging (MRI) demonstrated enlarged kidneys with enhanced echoes in the renal medulla and multiple visible cysts. The liver appeared enlarged with irregular surface contours, lobar disproportion, heterogeneous parenchymal signal intensity, and portal vein dilatation, suggesting possible cirrhosis (Figure 1). Both parents underwent abdominal ultrasonography and urinalysis, with all the results falling within normal limits.

### 3.2 Whole-exome sequencing and Sanger sequencing

**FIGURE 2:**
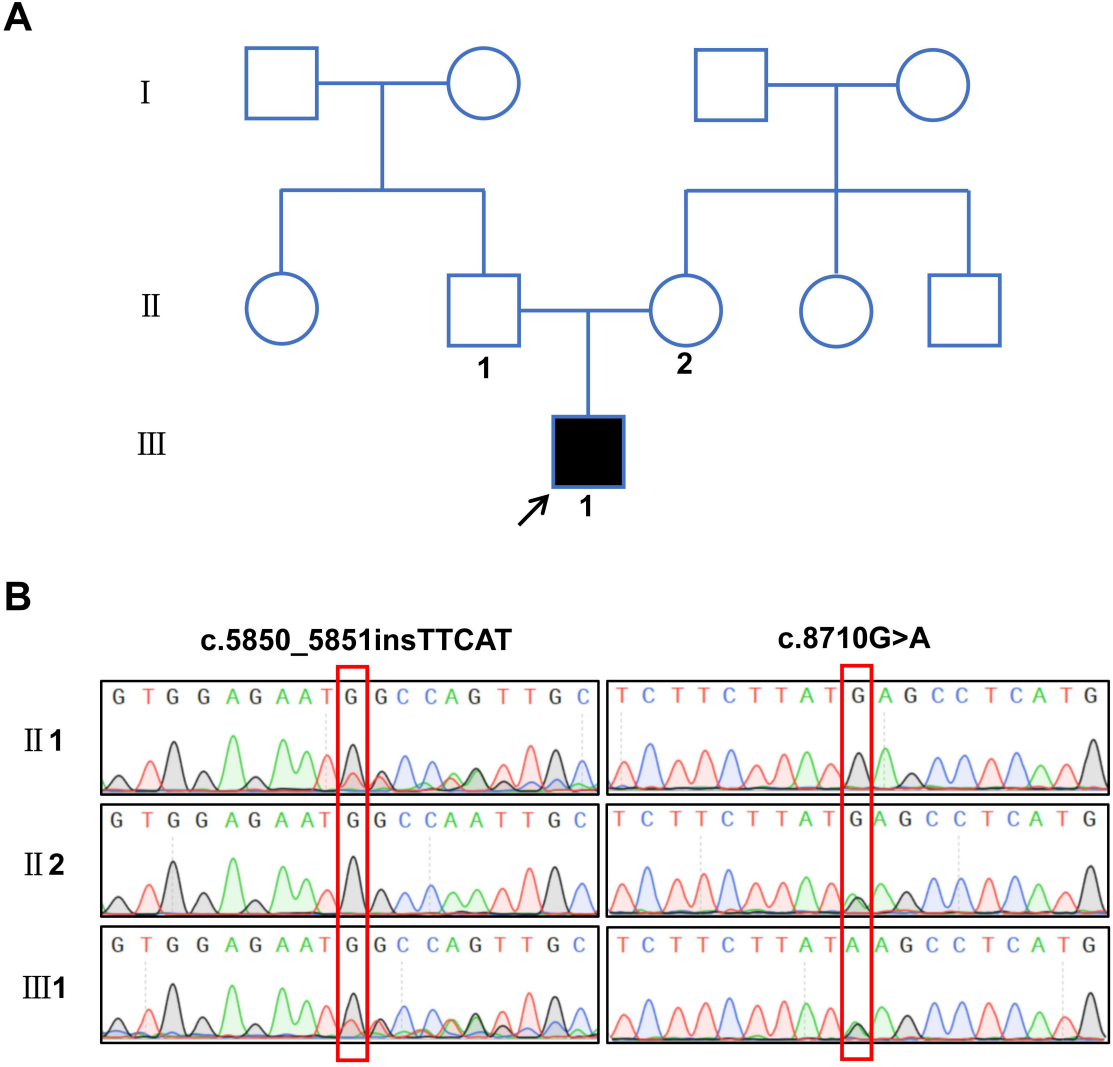
Family pedigree and sequencing results of the patient with polycystic kidney disease. **(A)** Pedigree of the proband’s family, the arrow shows the proband. **(B)** Sequencing results of compound heterozygous variant sites in the *PKHD1* gene.

Through whole-exome sequencing and validation by Sanger sequencing, the proband was found to carry a compound heterozygous variation in the *PKHD1* gene. The mutations included a frameshift mutation resulting from a multi-base insertion (c.5850_5851insTTCAT, p.Gly1951Phefs*25) and a missense mutation caused by a single-base substitution (c.8710G > A, p.Glu2904Lys). The father carried the c.5850_5851insTTCAT mutation, whereas the mother carried the c.8710G > A mutation (Figure 2). According to the ACMG classification, these two mutations were defined as pathogenic or likely pathogenic. Additionally, pathogenicity predictions using methods such as SIFT, PolyPhen-2, and Mutation Taster indicated that both variants were deleterious (Table 1).

**TABLE 1:** Clinical description and prediction of the pathogenesis of PKHD1 mutations.

Nucleotide change	Amino acid change	Zygosity	Mutation type	ACMG criteria	Mutation Taster	PROVEAN	SIFT	PolyPhen-2
c.5850_5851 insTTCAT	p.Gly1951Phefs	Het	Frameshift	P (PVS1 + PM2 + PP1)	Disease causing	NA	NA	NA
c.8710G > A	p.Glu2904Lys	Het	Missense	LP (PM1 + PM2 + PP1 + PP3)	Disease causing	−2.80 deleterious	0.01 damaging	0.999 probably damaging

Het, heterozygous; P, pathogenic; LP, likely pathogenic; NA, not available.

### 3.3 Pathogenesis analysis of *PKHD1* mutations

**FIGURE 3:**
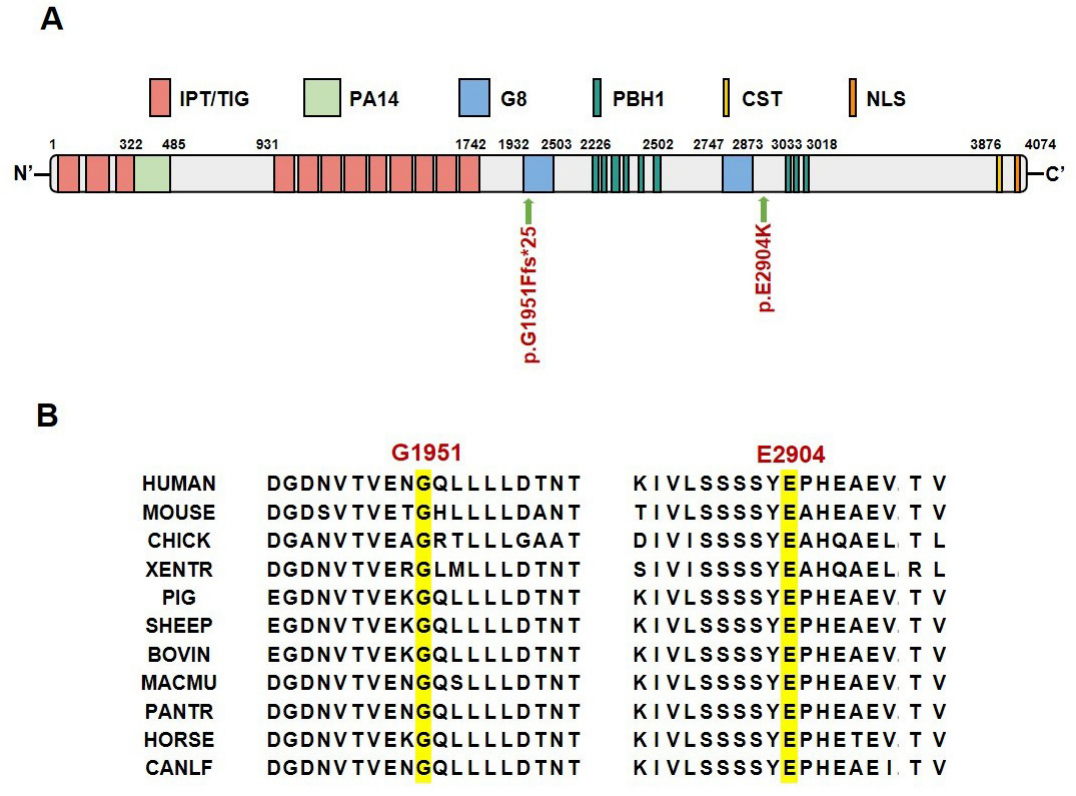
Location and conservation analysis of mutation sites. **(A)** Schematic diagram of FPC protein domains and mutation sites. **(B)** Conservation analysis of FPC mutated amino acid sites across different species. IPT/TIG, Ig-like, plexins, transcription factors/transcription factor Ig; PA14, anthrax protective antigen 14; G8, named for 8 conserved glycines; PbH1, parallel beta-helix repeats; CST, ciliary targeting sequence; NLS, nuclear localization signal.

**FIGURE 4:**
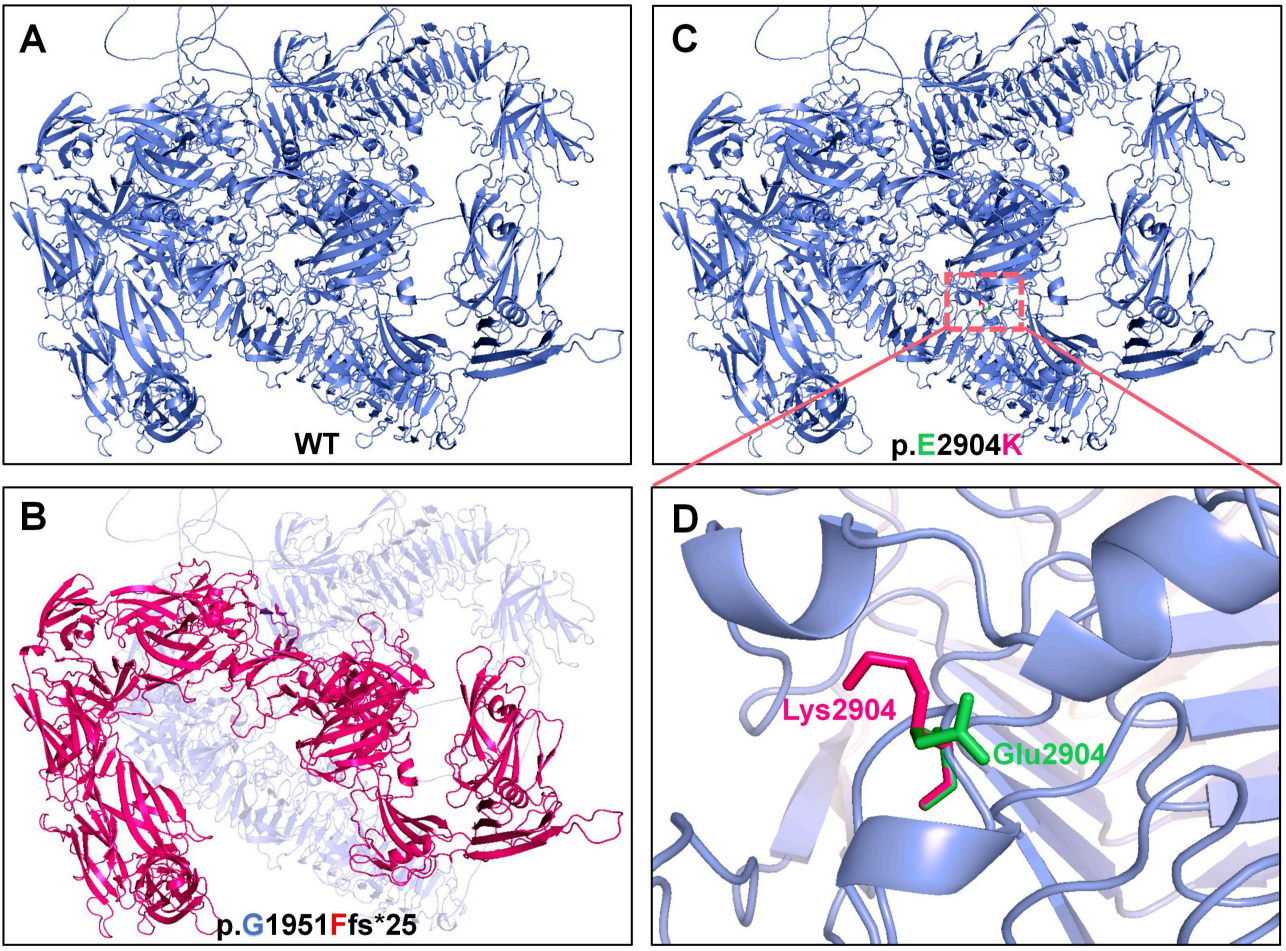
Structural prediction of wild-type and mutant FPC. **(A,B)** Wild-type structure (blue) and the truncated, destabilized structure predicted for the p.Gly1951Phefs*25 mutant (red). **(C,D)** Local wild-type conformation (green) and the altered conformation predicted for the p.Glu2904Lys mutant (red), highlighting the charge reversal from glutamate (E, negatively charged) to lysine (K, positively charged).

The *PKHD1* gene encodes the FPC protein, which comprises two core components. The first core component is the extracellular domain, which includes the N-terminal region and contains multiple domains of interest, such as IPT, PA14, G8, and PBH1. The second core component is the intracellular C-terminal domain, which has a ciliary-targeting sequence (Figure 3A). The p.Gly1951Phefs*25 mutation is located in the first G8 domain and leads to a shortened FPC protein containing only a partial extracellular domain, representing a loss-of-function mutation. The p.Glu2904Lys mutation is located in the extracellular domain and is adjacent to the second G8 domain. Conservation analysis of the FPC protein sequence across different vertebrate species revealed that both mutated amino acid positions, p.Gly1951Phefs*25 and p.Glu2904Lys, were highly conserved across species, suggesting that these mutations may be deleterious variations (Figure 3B). *In silico* structural modeling using AlphaFold3 predicted that these mutations would significantly affect the secondary structure of the FPC protein. Specifically, the p.Gly1951Phefs*25 mutation caused a frameshift mutation in the FPC protein, resulting in a shift of the protein-coding frame and premature termination 25 amino acids downstream of the mutation site, thereby shortening the protein length (Figures 4A, B). The amino acid Glu is a negatively charged polar amino acid, whereas Lys is a positively charged polar amino acid. The p.Glu2904Lys mutation may lead to a significant change in the charge properties that destabilize the protein. Furthermore, since glutamate acts as an α-helix breaker and lysine serves as an α-helix former, this mutation may also induce local secondary structural alterations. Consequently, the p.Glu2904Lys mutation is likely to alter the local conformation and impair the overall function of the FPC protein (Figures 4C, D).

### 3.4 Genotype-phenotype analysis of patients carrying *PKHD1* variants

**FIGURE 5:**
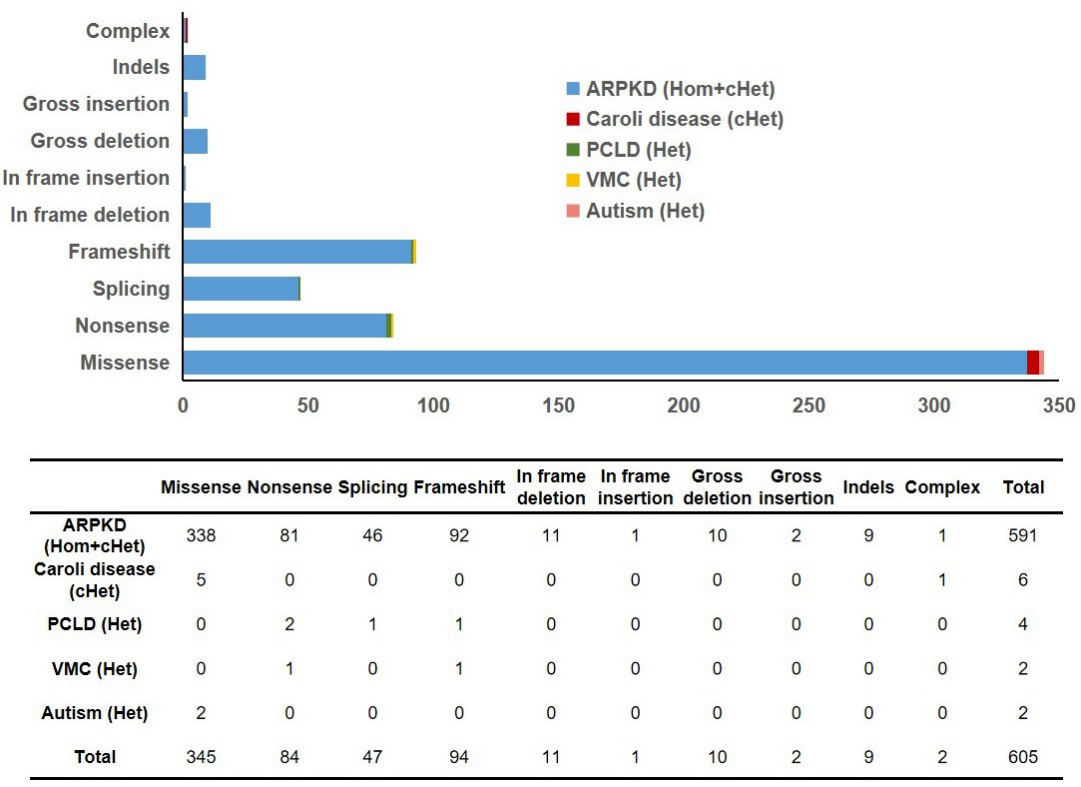
Summary of mutation types in 605 previously reported *PKHD1* variants. ARPKD, autosomal recessive polycystic kidney disease; PCLD, autosomal dominant isolated polycystic liver disease; VMC, von Meyenburg complexes; Hom, homozygous; cHet, compound heterozygous; Het, heterozygous.

A total of 605 *PKHD1* gene mutations and their corresponding clinical phenotypes were summarized (Figure 5 and Supplementary Table 1). Classification by mutation type revealed that missense mutations (345/605) were most prevalent, characterized by single-base-pair substitutions in coding regions. These mutations were followed in frequency by frameshift mutations (94/605), nonsense mutations (84/605), splicing mutations (47/605), in-frame deletions (11/605), large insertions (10/605), insertion-deletions (9/605), large deletions (2/605), complex mutations (2/605), and in-frame insertions (1/605). Although *PKHD1* deficiency is well-established in ARPKD, six *PKHD1* mutations have been identified in association with Caroli disease (CD, OMIM#600643), a rare autosomal recessive disorder characterized by segmental cystic dilatation of intrahepatic bile ducts, with an estimated occurrence rate of 1 per 1,000,000 live births (16). Notably, all three CD patients carried compound heterozygous mutations–two with missense mutation combinations and one with concurrent missense and complex mutations (Figure 5). Furthermore, four pathogenic mutations responsible for autosomal dominant isolated polycystic liver disease (PCLD) were identified (Figure 5). This condition shares identical hepatic cyst imaging and pathological features with ADPKD, but lacks clinically significant renal cysts (17). It is crucial to note that *PKHD1*-related disease exists on a continuum. The categorization of isolated hepatic disease is based on the absence of reported renal cysts at the time of publication and does not preclude later renal manifestations or subclinical renal pathology. Four PCLD patients all carried heterozygous loss-of-function mutations (frameshift, nonsense, or splice-site variants). Two additional mutations were associated with von Meyenburg complexes (VMC) (Figure 5), a rare type of ductal plate malformation, with no observed renal abnormalities in these heterozygous patients (18). Two other mutations showed potential associations with autism spectrum disorder, although further validation is required (Figure 5). These pathogenic variants were distributed throughout the *PKHD1* gene sequence without apparent mutational hotspots. The majority of patients exhibited ARPKD phenotypes with compound heterozygous *PKHD1* mutations. Only three homozygous missense mutation carriers manifested CD without renal involvement, whereas PCLD or VMC patients primarily carried heterozygous nonsense or frameshift mutations, and also lacked renal phenotypes (Figure 5). These findings indicate that the kidney may be less sensitive to *PKHD1* mutations compared to hepatic and biliary structures. Severe homozygous mutations may lead to renal phenotypes, whereas milder homozygous missense mutations or heterozygous states may result in attenuated or absent renal manifestations.

## 4 Discussion

Autosomal recessive polycystic kidney disease is a rare chronic kidney disorder characterized by the presence of cystic kidneys with numerous fluid-filled cysts in the renal parenchyma that continuously expand and progress throughout the lifetime of the patient (1, 19). This study reports a case of ARPKD with early-onset hepatic fibrosis caused by compound heterozygous mutations in the *PKHD1* gene. The proband was a 5-years-old boy who presented with significant hepatosplenomegaly and biochemical evidence of hepatic dysfunction, while renal function remained compensated despite enlarged kidneys with multiple cysts. Notably, this case exhibited two prominent features. The liver pathology manifested early and showed potential for progression to cirrhosis. Furthermore, both mutant alleles involved the G8 domains: p.Gly1951Phefs*25 is located within the first G8 domain, and p.Glu2904Lys lies adjacent to the second G8 domain, suggesting that the integrity of these domains may play a crucial role in the development and maintenance of the hepatobiliary system. This pattern resembles observations in some cases from Supplementary Table 1, where compound heterozygous or severe heterozygous mutations at certain loci appeared to correlate with significant liver involvement. The *PKHD1* gene encodes FPC, a type I transmembrane protein localized to primary cilia, involved in regulating cell proliferation, apoptosis, and polarity (20, 21). The two novel variants identified in the proband, c.5850_5851insTTCAT (frameshift) and c.8710G > A (missense), are highly conserved in vertebrates, suggesting potential deleterious effects. Structural analysis based on AlphaFold predictions indicated that p.Gly1951Phefs*25 leads to premature translational termination and loss of the intracellular C-terminal domain, potentially disrupting the interaction with polycystin-2 (22, 23); p.Glu2904Lys may cause local charge reversal and alter α-helical propensity, thereby changing conformation and weakening extracellular matrix adhesion. However, these structural inferences are derived from *in silico* modeling, and further biological experiments are required to validate the pathogenicity of these mutations.

A total of 605 *PKHD1* variants were collated in this study. Genotype-phenotype analysis revealed a trend: the vast majority of patients exhibited the ARPKD phenotype associated with compound heterozygous *PKHD1* mutations. Additionally, three patients with compound heterozygous missense mutations presented with Caroli disease without renal involvement; four patients with PCLD and two patients with VMC primarily carried heterozygous nonsense or frameshift mutations and showed no renal phenotype at the last evaluation. These findings suggest that the hepatobiliary system may be more sensitive to specific forms of *PKHD1* deficiency, while severe renal cystic phenotypes tend to occur in individuals with biallelic loss-of-function mutations. Severe heterozygous mutations (nonsense/frameshift) or mild compound heterozygous missense mutations may be sufficient to cause hepatobiliary abnormalities, which may be related to the crucial role of FPC in ductal plate remodeling and the lack of compensatory mechanisms (24). Conversely, severe renal cystic phenotypes typically require biallelic loss-of-function mutations, likely because polycystin-1/polycystin-2 may partially compensate for FPC dysfunction during collecting duct development (25). In this study, the proband developed liver fibrosis/cirrhosis at 5 years of age while renal function remained compensated, further supporting the hypothesis that the hepatobiliary system may be more sensitive to FPC dysfunction. However, these observations are based on cross-sectional data presented in Supplementary Table 1, without statistical testing or longitudinal adjustment, and lack estimates of population prevalence or risk; therefore, they should be regarded as hypothesis-generating findings rather than definitive biological principles.

This study systematically outlines the mutation types and corresponding phenotypes of Caroli disease, PCLD, and VMC in a relatively large cohort. The data indicate that specific mutation combinations or heterozygous loss-of-function variants may be sufficient to cause hepatobiliary abnormalities, which may reflect the critical role of FPC in ductal plate remodeling and its limited compensatory capacity (26–29). Based on these preliminary findings, it is proposed that regular liver ultrasound monitoring for *PKHD1* heterozygous carriers could be considered as a potential clinical consideration. However, it is acknowledged that current evidence is limited, the number of such cases is small, prevalence and risk estimates are lacking, and existing clinical guidelines do not recommend routine screening for asymptomatic carriers. Validation in larger, longitudinally followed cohorts is needed to untangle organ-specific susceptibility and inform risk-stratification strategies. Notably, the proband’s father carries the *PKHD1* frameshift mutation c.5850_5851insTTCAT (p.Gly1951Phefs*25) but showed no abnormalities on abdominal ultrasound or urinalysis, indicating no clinically detectable hepatobiliary or renal phenotype in adulthood. Possible explanations include developmental or genetic modifiers that attenuate phenotypic expression; mutation-site-dependent functional impact; limitations of conventional imaging in detecting subtle or early lesions; and age-dependent phenotypic manifestation. Therefore, the mere presence of a truncating variant does not predict disease occurrence, emphasizing the need for cautious interpretation in genetic counseling and consideration of longitudinal familial assessment.

This study expands the mutational spectrum of *PKHD1*-related disorders, provides genotype-phenotype observations based on a large-scale variant dataset, and offers references for genetic counseling and prenatal diagnosis for the studied family. Major limitations include: the relatively small number of reported cases with so-called isolated liver phenotypes (CD, PCLD, VMC), limiting statistical power; reliance on *in silico* structure-function inferences without functional experimental validation; the dichotomous classification of phenotypes failing to fully capture the disease continuum, where renal involvement may be delayed or subclinical; and the integration of data from heterogeneous sources with incomplete clinical information or short follow-up in the genotype-phenotype analysis dataset. Future studies should employ standardized methods to recruit larger cohorts, conduct functional experiments, and perform lifelong clinical monitoring to elucidate the molecular basis and clinical implications of organ-specific susceptibility.

## 5 Conclusion

In summary, this study expands the mutational spectrum of ARPKD, and analysis of reported *PKHD1* variants suggests a potential correlation between mutation severity and phenotypic expression, with hepatobiliary manifestations may be associated with a milder mutational burden compared with renal phenotypes. Our findings underscore the utility of genetic diagnosis for counseling and indicate the need for further investigation to clarify the true organ-specific vulnerability.

## Data Availability

The datasets presented in this study can be found in online repositories. The names of the repository/repositories and accession number(s) can be found in this article/Supplementary material.

## Footnotes

1
http://www.ACMG.net/

2
https://primer3.org/

3
https://www.ncbi.nlm.nih.gov/

4
http://www.bioinformatics.org/strap/

5
https://alphafoldserver.com/

6
http://www.pymol.org
